# Relationship between a Weighted Multi-Gene Algorithm and Blood Pressure Control in Hypertension

**DOI:** 10.3390/jcm8030289

**Published:** 2019-02-28

**Authors:** Pamela K. Phelps, Eli F. Kelley, Danielle M. Walla, Jennifer K. Ross, Jerad J. Simmons, Emma K. Bulock, Audrie Ayres, Monica K. Akre, Ryan Sprissler, Thomas P. Olson, Eric M. Snyder

**Affiliations:** 1 Medical Center, University of Minnesota, Fairview, Minneapolis, MN 55455, USA; pphelps2@fairview.org (P.K.P.); dwalla1@fairview.org (D.M.W.); jross13@fairview.org (J.K.R.); jsimmon7@fairview.org (J.J.S.); ebulock1@fairview.org (E.K.B.); aayres1@fairview.org (A.A.); 2 School of Kinesiology, University of Minnesota, Minneapolis, MN 55455, USA; kelle833@d.umn.edu; 3 Geneticure, Inc., Rochester, MN 55902, USA; monica@geneticure.com (M.K.A.); ryans1@email.arizona.edu (R.S.); olson.thomas2@mayo.edu (T.P.O.); 4 University of Arizona Genomics Core, University of Arizona, Tucson, AZ 85705, USA; 5 College of Medicine, Mayo Clinic, Rochester, MN 55905, USA

**Keywords:** hypertension, blood pressure, genetics, pharmacogenetics, pharmacotherapy, treatment

## Abstract

Hypertension (HTN) is a complex disease with interactions among multiple organ systems, including the heart, vasculature, and kidney with a strong heritable component. Despite the multifactorial nature of HTN, no clinical guidelines utilize a multi-gene approach to guide blood pressure (BP) therapy. Non-smokers with a family history of HTN were included in the analysis (*n* = 384; age = 61.0 ± 0.9, 11% non-white). A total of 17 functional genotypes were weighted according to the previous effect size in the literature and entered into an algorithm. Pharmacotherapy was ranked from 1–4 as most to least likely to respond based on the algorithmic assessment of individual patient’s genotypes. Three-years of data were assessed at six-month intervals for BP and medication history. There was no difference in BP at diagnosis between groups matching the top drug recommendation using the multi-gene weighted algorithm (*n* = 92) vs. those who did not match (*n* = 292). However, from diagnosis to nadir, patients who matched the primary recommendation had a significantly greater drop in BP when compared to patients who did not. Further, the difference between diagnosis to current 1-year average BP was lower in the group that matched the top recommendation. These data suggest an association between a weighted multi-gene algorithm on the BP response to pharmacotherapy.

## 1. Introduction

Hypertension (HTN) is a major preventable contributor to cardiovascular disease (CVD) and accounts for approximately 360,000 of the 2.4 million (14%) annual deaths in the United States [1,2,3]. High blood pressure represents an estimated $51 billion in direct costs to the United States health care system [4]. Furthermore, HTN is known to lead to myocardial infarction, stroke, renal failure, and death if not detected early and treated appropriately, resulting in an estimated $113 billion total (direct and indirect) annual cost [1,5]. With the advent of improved diagnostic techniques, increased rates of health care utilization and screening, and the increasing age of the population, a continual upward trend in this expenditure is expected [6]. Despite the prevalence of HTN, control rates are generally poor [7,8]. While 70% of HTN patients are treated, only half of those achieve blood pressure (BP) control (BP < 140/90 mmHg) [9]. These poor control rates, despite high treatment rates, suggest that the efficacy of BP therapy goes beyond adherence. This is further supported by detailed studies that have demonstrated an average effective rate of 50% for each common class of BP medication (diuretic, ACE-inhibitors, angiotensin-II receptor blockers, beta-blockers) [10], even when adherence is confirmed. Additionally, most HTN pharmacotherapies demonstrate a bell-curve response, such that a majority of patients have a reduction or no change in BP, but 10–20% of patients demonstrate an increase in BP [11,12].

Hypertension has a strong heritable component, estimated at approximately 50%, with current studies suggesting the responsiveness to HTN therapy may be heritable as well [12,13,14,15,16]. It has been shown that the risk of developing HTN is increased two-fold for each first degree relative with HTN [13]. Furthermore, when compared to sons of normotensive individuals, sons of HTN patients have a 10 mmHg higher systolic and diastolic BP, independent of dietary Na^+^ intake [13,17]. Additionally, the correlation for risk of HTN development is 55% in monozygous twin siblings, up to 40% in dizygous twins, and as low as 20% for non-twin sibling pairs [18,19,20]. Collectively, these data demonstrate a clear genetic component to HTN development. While both genome-wide association studies and candidate gene studies have demonstrated that HTN monotherapy effectiveness may be improved using genetics to guide therapy [14,15], few studies have sought to identify the impact of genotypes, important in BP regulation and pharmacotherapy response, on multiple drug classes simultaneously [11,15].

In an effort to control HTN, given the potential for ineffective monotherapy, the current standard of care is to “layer” BP drugs (i.e., increasing dosage to the maximally tolerated dosage then adding a second HTN therapy on top of the first therapy). If guideline-directed blood pressure goals continue to be elusive (following layering of several classes of drugs at the maximally-tolerated doses), the process is reinitiated with a different combination of drugs, different classes of drugs, different drug options within a given class of drugs, different dosages or all of the above. This trial-and-error standard of care is clearly not optimal and presents consequences, such as increased side effects, costs to the patient, increased health care service utilization, and reduced quality of life [21,22]. Furthermore, previous work has demonstrated an 80% reduction in medication adherence for each new drug prescribed [23].

Research examining genetic determinants to HTN therapy response have identified β_1_-adrenergic receptor (ADRB1), β_2_-adrenergic receptor (ADRB2), cytochrome P450 2D6 (CYP2D6), lysine deficient protein kinase (WNK), sodium-chloride symporter (SLC12A3), alpha subunit of the epithelial sodium channel (SCNN1A), alpha-adducin (ADD1), renin, angiotensin, angiotensin-converting enzyme (ACE), and angiotensin receptor variants as playing key functional roles in response to HTN therapy [12,16,24,25,26]. Genetically guided monotherapies based on these functional variants result in an approximate 4–10 mmHg greater drop in mean arterial pressure (MAP) [12,25,26,27,28,29,30,31,32,33,34,35,36]. Furthermore, genotype association studies have demonstrated improved clinical outcomes when the functional genotype is taken into consideration [11,12,16,24]. These clinical outcomes include: improved K^+^ and Cl^−^ excretion, decreased adverse events (myocardial infarction, stroke, coronary heart disease) and decreased mortality risk [37,38,39,40,41]. Despite the clear promise of genetically-guided therapy for BP control, clinical care settings are slow to adopt this approach.

Collectively, these previous studies demonstrate that genetic variations in proteins responsible for BP modulation may be responsible, in part, for the variability of HTN therapy effectiveness. To date, most research on BP therapies efficacy has been monogenic and have not adequately taken into account the multi-organ and multi-system integrative nature of HTN. Furthermore, while emerging data has employed the use of a simple algorithm, taking into account the total number of functional genotypes within an organ system, to predict BP response to therapy, pilot work by our group has demonstrated that this approach alone does not accurately predict BP response. Indeed, some genotypes demonstrated functionality with respect to BP response to HTN therapy whereas some genotypes demonstrated functionality with respect to BP control using a <140/<90 mmHg cutoff. These differences suggest that a more complex and logical algorithm is needed to more accurately predict BP response to HTN therapy [42]. This lack of efficacy when genotypes are considered in isolation is likely due to the numerous genes that need to be considered to guide therapy, simultaneously and with gene-gene interactions, and the pathways in the cardiorenal axis responsible for BP control [16,43,44]. In order for HTN pharmacogenetics to be effective, these genes must be weighted based on their effect size and importance in BP regulation, in order to adequately predict BP response. Therefore, in the present study, we assessed 17 genotypes within the heart, vasculature, and kidney on the BP response to therapy. This builds on previous research through the weighting of each genotype by their effect size (ΔBP with treatment for a functional variant on target therapy vs. ΔBP for a non-functional variant on the same therapy) as determined by previous literature [24,45,46,47,48,49,50,51,52,53,54,55,56,57,58,59,60]. After weighting, each genotype was entered into an algorithm that assessed pairing (e.g., homozygosity of functional ADRB1 allele is responsive to β-blockade, heterozygosity is less responsive, and homozygosity of non-functional allele is least responsive) within and between organ systems to predict the pharmacotherapy with the greatest impact for each individual patient.

## 2. Methods

### 2.1. Study Design

This study was designed and performed in a primarily retrospective manner and included patients within Fairview Health Services (clinicaltrials.gov identifier: NCT02524873). Patients provided DNA for genetic analysis, and a detailed medical chart review was performed between 30 November 2015 and 30 November 2018 with a focus on the present study in the 12-months prior to DNA collection. All patients provided written informed consent prior to enrollment and data collection (University of Minnesota IRB# 00000756). Patients included non-smokers with a family history of HTN and a history of BP control (<140/<90 for two consecutive office measures). The primary outcome variables included an office BP (averaged from one year of patient visits) and level of BP control using traditional guidelines for patients with and without diabetes (<140/<90) and Systolic Blood Pressure Intervention Trial (SPRINT) guidelines (<120/<80). Primary chart measurements included systolic blood pressure (SBP) and diastolic blood pressure (DBP), and mean arterial pressure (MAP) was calculated. Additional measures included current medications, a detailed hypertension medication history, side effects from previous medications, and medication adherence. Three-years of medical chart data were examined at six-month intervals for BP and medication history to quantify the number of medications needed to obtain BP control and BP control history. Current and past pharmacotherapies were also assessed via chart review. Additional variables included a change in BP measurements from diagnosis to the most recent one-year chart review period.

### 2.2. Subjects

All patients enrolled in the study were HTN patients who had their BP under control for at least six months and who had an HTN diagnosis for at least one year. In addition to BP history, demographic information collected included age, sex, height, weight, race, and ethnicity. Inclusion criteria for the study included: 20–85 years of age, patient on the same class/classes of blood pressure medication for a minimum of six months (a change in dosage, frequency or specific medication was accepted as long as there had been no changes to the class/classes of medications prescribed), a body mass index (BMI) between 19 and 45 kg/m^2^, patient prescribed and taking one of the following classes of medications alone or in combination: diuretics (thiazide or thiazide-like), ACE inhibitors (ACEI), angiotensin receptor blocker (ARB), beta-blockers, and Ca^2+^ channel blockers. Subjects were excluded from participation if one or more of the following conditions were met: a diagnosis of secondary HTN or a complication of pregnancy, currently prescribed and taking any additional class of medication(s) for HTN not included in the inclusion criteria list, and SBP > 140 or DBP > 90 documented within the immediate six months prior to the study visit.

### 2.3. Cell Collection and Genotyping

Two buccal swabs were collected for each patient. Cells were first collected via a buccal brush by swabbing the inside of their right cheek repeatedly (for fifteen seconds using moderate pressure) (A-swab). The patient then deposited the swab in 750 µL of lysis buffer consisting of 50 mM Tris pH 8.0, 50 mM EDTA, 25 mM Sucrose, 100 mM NaCl, and 1% SDS to lyse the cells and stabilize DNA during transit prior to extraction. This process was repeated with the left cheek (B-swab) to ensure adequate cell collection necessary to achieve a minimum yield of 500 ng total gDNA for downstream genotyping. Subsequent lysate from buccal swabs was used in DNA isolation via Qiagen DNeasy isolation kits according to the manufacturer’s recommended specifications (Qiagen, Hilden, NorthRhine-Westphalia, Germany). Patient isolated DNA was then assayed for 17 functional alleles in 11 genes with known functionality in the heart, kidney, and vasculature: (1) *ADRB1* (*rs1801252* and *rs1801253*), (2) *ADRB2* (*rs1042713* and *rs1042714*), *SCNN1A* (*rs2228576*), alpha-adducin (ADD1, *rs4961*), *SLC12A3* (*rs1529927*), (3) angiotensin (AGT, *rs699*, *rs5051*, and *rs7079*), renin (REN, *rs12750834*), WNK1 (*rs1159744*, *rs2107614*, and *rs2277869*), angiotensin-converting enzyme (ACE, *rs1799752*), angiotensin receptor (AGTR1, *rs5186*), and cytochrome P450 2D6 (CYP2D6*4, *rs3892097*). With the exception of the ACE insertion/deletion (indel) genotype, all genotype polymorphisms were quantified using a two-step process beginning with a multiplex PCR, directly followed by a single base extension (SBE) reaction. The products of the SBE reaction were pooled and subsequently flown on a genomic mass spectrometer (Agena MassARRAY system, San Diego, CA, USA) to generate individual genotypes. The ACE indel status was assessed using a standard fluorescently labeled PCR primer set and protocol, followed by fragment analysis via a 3730 DNA analyzer (Applied Biosystem, Foster City, CA, USA). Pre-characterized Coriell cell line DNAs consisting of all possible genotype combinations were run in parallel for each single nucleotide polymorphism (SNP) as controls. All genotype data in aggregate was also used to compute population allele frequencies which were then confirmed against known existing frequencies in publically available databases (ExAC browser, 1000 Genomes project, GO-exome sequencing project, and TOPMED).

### 2.4. Mathematical Prediction of Drug Responsiveness

This study builds on our previous work in that we use weighted scoring for each genotype to develop a weighted multi-gene algorithm to determine if a patient would respond to one drug over another. Functional genotypes affecting BP response to pharmacotherapy as identified by current literature were included in the mathematical prediction, including 17 SNPs in 11 genes (*ADRB1*, *ADRB2*, *CYP2D6*, *WNK*, *SLC12A3*, *SCNN1A*, alpha-adducin, renin, angiotensin, ACE, and the angiotensin receptor). These genotypes were weighted according to previous effect size in the literature (∆BP when on the target therapy vs. not for functional and non-functional genotypes) as well as the number and quality of peer-reviewed papers supporting each functional variant. Specifically, the genotypes that had the largest effect (plasma drug levels or ΔBP with treatment in the functional genotype group vs. without treatment) were *CYP2D6*, both sites of *ADRB1*, position 27 *ADRB2*, one site of WNK (*rs227869*), the alpha-adducin genotype, and two genotypes for angiotensin (*rs5051* and *rs699*) (Table 1). Following this, the genotypes that seem to had a lower to moderate effect (ΔBP in the previous literature) based on aggregate data included position 27 of the *ADRB2*, two sites for *WNK* (*rs1159744* and *rs2107614*), *SLC12A3*, *SCNN1A*, renin, ACE, angiotensin (*rs7079*), and the angiotensin-II receptor. In addition to weighing based on the ΔBP from the previous literature, genotypes were also weighted based on the strength of the peer-reviewed literature. Genotypes with more peer-reviewed papers were given a higher scoring priority with the assumption that there is more agreement in the functionality, as also summarized in Table 1. After weighting, each genotype was entered into an algorithm that assessed pairing (e.g., homozygosity of functional ADRB1 is responsive to β-blockade, heterozygosity is less responsive, and homozygosity of non-functional is least responsive) within and between organ systems to predict the pharmacotherapy with the greatest impact for each individual patient. Each pharmacotherapy predicted to be beneficial was ranked from 1–4 as most to least likely to be effective for that patient based on the algorithmic assessment of individual patient’s genotypes.

### 2.5. Data Analysis

All data were coded for statistical analysis (i.e., drug classes and genotypes coded numerically according to functionality) and were analyzed with SPSS version 21. Normality of the data was assessed using Levene’s test prior to statistical analysis to assess equality of variance to ensure appropriate statistical methodology. Descriptive statistics were computed (age, height, weight, BMI, etc.) and reported as mean ± standard deviation (SD). Post-hoc correction for univariate analysis of the variance was conducted using Bonferroni analysis. Ordinary least squares regression via univariate modeling was used to estimate the magnitude of linearity between drug classes that yielded the best blood pressure control and those which were mathematically predicted based on the sum of genotypes for the subject. All statistical analyses were considered significant at an alpha level of 0.05.

## 3. Results

Three hundred and eighty-four subjects met the inclusion criteria, were genotyped, and had complete chart data for review. The distribution of patients currently on the target drug class of each polymorphism is presented in Table 2. Patients were grouped into those who were on the current prescription for drug therapy which matched to the top recommendation provided from the algorithm vs. those who did not match (Table 3). There were no differences in patient age, smoking status, drinking status, amount of exercise or self-reported health status. Patients who matched their top drug recommendation using the multi-gene weighted algorithm differed significantly in height, weight, and BMI compared to patients who did not match their top drug recommendation (Table 4).

**Figure 1:**
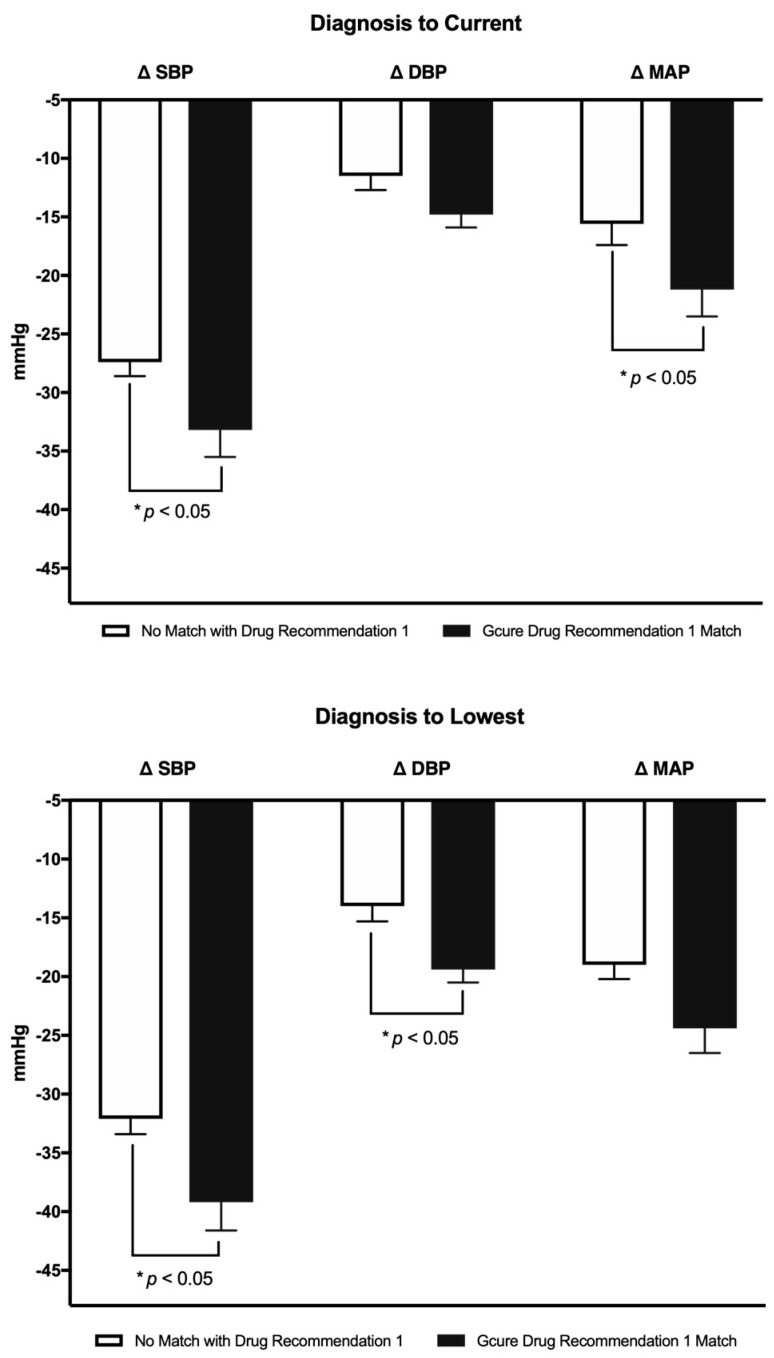
Change in Blood Pressure for Patients Whose Therapy Matched the Primary Recommendation Compared to Patients Whose Therapy did not. Panels depict the change in systolic blood pressure (SBP), diastolic blood pressure (DBP), and mean arterial blood pressure (MAP) from diagnosis to the lowest measurement and from diagnosis to current one-year average between patients whose therapy matched the genetically determined primary drug class compared to patients whose therapy did not match. * *p* < 0.05.

There was no difference in initial BP at diagnosis between groups matching the top drug recommendation using the multi-gene weighted algorithm compared with those who did not match their top drug recommendation. Further, there was no difference between groups in the percent of patients under BP control as defined by Joint National Committee (JNC) and SPRINT guidelines (Table 5). However, from diagnosis to nadir, patients who matched the primary recommendation had a significantly greater drop in BP when compared to patients who did not (∆SBP = −39.2 ± 2.4 vs. −32.1 ± 1.3 mmHg, ∆DBP = −19.4 ± 1.1 vs. −14.0 ± 1.3 mmHg, ∆MAP = −24.4 ± 2.1 vs. −19.0 ± 1.2 mmHg, respectively, *p* < 0.05 for SBP and DBP). Further, the difference between diagnosis to current 1-year average BP was lower in the group that matched the top recommendation (∆SBP = −33.2 ± 2.3 vs. −27.4 ± 1.2 mmHg, ∆DBP = −14.8 ± 1.1 vs. −11.5 ± 1.2, ∆MAP = −21.2 ± 2.3 vs. −15.6 ± 1.8, respectively, *p* < 0.05 for SBP and MAP) (Figure 1).

In order to better assess the effect of the algorithm on drug response, we calculated the change in BP and HTN control rates in a sub-group of patients on monotherapy (Table 6). In this group, if a patient matched the primary drug recommendation, they tended to have a lower change in BP from the time of diagnosis (~5 mmHg, on average), but this was not statistically significant. Interestingly, patients who matched the first drug recommendation, and were only on one pharmacotherapy, were ~50% more likely to have their BP under control using the newer SPRINT guidelines (27% control vs. 18% control, for those patients who were on only one medication and matched the first algorithmic recommendation compared to those who did not match, respectively).

Because many patients are on more than one pharmacotherapy, we also assessed the response to treatment for patients who matched the drug recommendation one or drug recommendation two from the algorithm. From this analysis, we found that there was no significant difference in the drop in blood pressure between patients matching recommendation one or recommendation two, but that patients who did match were slightly more likely to have their BP under control with the newer SPRINT guidelines (27% vs. 22% for those matching recommendation one or two vs. those who did not match, respectively) (Table 7).

## 4. Discussion

In this study, we assessed the BP values and hypertension status between HTN patients who matched the top drug recommendation using a multi-gene scientifically-weighted algorithm and those who did not match. Our data suggest that few patients’ (24%) prescribed therapy matched their recommended therapy using the weighted algorithm. However, patients who matched the primary recommendation had a significantly greater drop in BP (SBP, DBP, and MAP), from the time of diagnosis to current clinical data, when compared to patients who did not match. Further, the difference between diagnoses to the current one-year average BP was lower in the group that matched the top recommendation. Additionally, for patients on monotherapy, those matching the primary algorithmic drug recommendation were ~50% more likely to have their BP under control using the newer SPRINT guidelines. Interestingly, subjects who matched the first drug recommendation were heavier and tended to exercise less, suggesting the possibility that the findings are rather conservative. Collectively, these data suggest that a multi-gene scientifically-weighted algorithm is more effective in determining BP response and guiding HTN therapy than monogenic therapy alone.

Hypertension is a highly multi-factorial disease modulated by multiple susceptibility genes, suggesting a large genetic determinant to HTN therapy response. Research examining genetic determinants to HTN therapy response have identified β_1_-adrenergic receptor (*ADRB1*), β_2_-adrenergic receptor (*ADRB2*), cytochrome P450 2D6 (*CYP2D6*), lysine deficient protein kinase (WNK), sodium-chloride symporter (*SLC12A3*), alpha subunit of the epithelial sodium channel (*SCNN1A*), alpha-adducin (*ADD1*), renin, angiotensin, angiotensin-converting enzyme (ACE), and angiotensin receptor variants as playing functional roles in response to HTN therapy [12,16,24,25,26]. The influence of multiple genes on HTN therapy response suggests that the multi-gene therapies, based on the strength of previous peer-review studies, would be more effective than monogenic therapy.

A primary pathway responsible for the modulation of fluid balance and BP maintenance is the renin-angiotensin-aldosterone system (RAAS). Within the RAAS, renin and angiotensin-converting enzyme (ACE) are integral in the formation of angiotensin II (Ang II), the primary product of RAAS and a potent vasoconstrictor [78]. Angiotensin II has several functions in the cardiovascular system, kidney, sympathetic nervous system, and adrenal cortex important in BP maintenance [79]. The research focused on genetic variations of the ACE-inhibitor (ACEI), Ang I, and the Ang II receptor suggests that the renin-dependent mechanisms are involved in approximately 70% of HTN [78]. Specifically, the deletion variant of ACE (*rs7079*), the C variant of Angiotensin (*rs699*), the C variant of the angiotensin-II receptor (*rs5186*), and the C-5312T variant of REN (*rs12750834*) have shown improved response to ACEI and Ang II receptor antagonism and decreased risk of stroke and coronary heart disease with therapy [27,28,29,30,69].

Additional regulators of RAAS products, integral in BP homeostasis, are the beta-adrenergic receptors (*ADRB1*, *ADRB2*). The beta-adrenergic receptors are found primarily in the heart and juxtaglomerular cells of the kidney where they mediate important cardiovascular responses and RAAS activity [80]. Stimulation of the beta-adrenergic receptors in the kidney induces renin release activating RAAS [81,82]. Aldosterone, a downstream product of RAAS, preferentially increases renal tubular luminal Na^+^ transport and plays a functional role in fluid volume and BP regulation [83]. Furthermore, SBP and DBP are impacted by RAAS activity and reflect Na^+^ retention [83]. The role the beta-adrenergic receptors play in RAAS activation demonstrates the functional role they play in HTN. Currently, BP response to beta-blockers has focused primarily on genetic variants of *ADRB1* and *ADRB2*. Specifically, the Ser49 and Arg389 of *ADRB1* (*rs1801252* and *rs1801253* respectively) and the Arg16 and Glu27 of *ADRB2* (*rs1042713* and *rs1042714* respectively) have demonstrated improved responsiveness to and decreased adverse event risk and mortality with beta-blockade [31,32,33,37,38].

Additional mechanisms of action of aldosterone and Ang II is the modulation of Na^+^ reabsorption and K^+^ secretion through direct epithelial sodium channel (ENaC) stimulation [79]. The epithelial sodium channel (ENaC) is an ion channel critical in the maintenance of extracellular fluid volume, BP, and sodium homeostasis via Na^+^ reabsorption and H_2_O diffusion across the apical membrane of renal nephrons [84,85]. The research suggests that *SCNN1A* is the primary regulator of Na^+^ flux through ENaC and genetic variations of *SCNN1A* are associated with BP modulation [86,87]. While ENaC regulates Na^+^ transport across the luminal plasma membrane, Na-K-ATPase regulates Na^+^ transport across the basolateral plasma membrane. This suggests that the complex interplay between the Na-K-ATPase and ENaC is an important pathway in fluid volume regulation [34]. The primary regulator of this complex is adducing, which is responsible for the expression and maximum velocity of Na-K^−^ATPase and subsequently increases renal tubular Na^+^ reabsorption [88,89,90]. The research has demonstrated genetic variants of the alpha-adducin subunit (*ADD1*) influencing HTN development risk and diuretic therapy responsiveness [34]. Specifically, the T variant of alpha-adducin (*rs4961*) has been shown to be more responsive to a diuretic and to halve the risk of myocardial infarction and stroke with BP control [34,40].

Other pathways in fluid volume and BP regulation include ion co-transporters that modulate Na^+^ reabsorption in the kidneys, including members of the *SLC12* family of ion transporters and have vital roles in regulating electrolyte transport and BP [91]. These transporters are targets for thiazide-diuretic and loop-diuretic therapy for BP control. The *SCL12A* is a kidney-specific isoform expressed in the distal convoluted tubule, and the C variant of *SLC12A3* (*rs1529927*) has been demonstrated to be more responsive to diuretic therapy [34,91]. Furthermore, the research has identified lysine deficient protein kinases (WNKs) as upstream regulators of these pathways [36]. The research has demonstrated that *WNK1* (the kidney-specific isoform) plays a regulatory role in electrolyte transport across renal membranes and epithelia, suggesting a functional role in BP maintenance [35,92,93]. Further, genetic variations of *WNK1* have been associated with an ~5–6 mmHg variability in BP response to hydrochlorothiazide (*rs2107614*, *rs1159744*, and *rs2277869*) [12,35,36]. This relationship is likely attributable to the improved Na^+^ and Cl^−^ excretion associated with these variants [41].

To further understand the influence of pharmacotherapy on BP response, it is necessary to consider pharmacokinetics. The liver is the principal site of drug metabolism, primarily by a class of proteins called Cytochrome P450 (CYP). To date, 12 human CYPs have been identified, including *CYP2D6* [94]. While *CYP2D6* only accounts for 2–8% of all hepatic CYPs, it is responsible for the metabolism of approximately 25% of clinically used drugs [94,95]. Two common variants of the *CYP2D6* have been identified as either fully functional (CYP2D6*1) or non-functional (CYP2D6*4). These genetic variations of CYP2D6 have been associated with responsive beta-blocker therapy through differential metabolism. The research has demonstrated CYP2D6*4 carriers to have an up to 5-fold increase in plasma drug levels after beta-blocker therapy [25]. Despite this dramatic difference in plasma beta-blocker levels, most studies have demonstrated no effect on BP as an outcome variable.

Cumulatively, these data suggest that HTN is a complex, multi-factorial disease comprised of multiple organ systems and pathways. While there is a push towards a “personalized medicine” approach to BP pharmacotherapy, the current clinical treatment strategy is based on a set algorithm which bases its recommendations on drug class and does not differentiate between drug classes [96]. As a result, different clinical algorithms preferentially recommend certain drug classes over others [96]. Furthermore, commonly used algorithms categorize their patients by age, ethnicity, hypertensive stage, and other clinical characteristics (i.e., diabetes, coronary disease, etc.) to guide HTN patient pharmacotherapy [96]. To date, clinical algorithms fail to fully incorporate genetics-based pharmacotherapy for BP control.

The influence of the aforementioned genetic variants on BP response to therapy clearly demonstrates the impetus for a multi-gene approach to pharmacotherapy. This study has been built on previous monogenic studies with the inclusion of multiple genotypes in multiple organ systems, and on previous multi-gene studies with the implementation of a weighted, multi-gene algorithm for determining BP response to therapy. The current study demonstrated a significantly greater drop in BP in patients who matched the primary recommendation when compared to patients who did not. Further, the difference between diagnoses to current 1-year average BP was lower in the group that matched the top recommendation. These data suggest an algorithm that is weighted based on the strength of previous peer-review studies, is multi-gene in nature and predictive of the BP response to pharmacotherapy compared to the current standard of care, however, prospective trials are needed.

## 5. Conclusions

The purpose of this study was to assess seventeen common genetic variants in the liver (drug metabolizing enzyme), cardiac, vascular, and renal systems to determine the effectiveness of a weighted multi-gene algorithm on predicting BP response to therapy. We demonstrated an association between a scientifically-weighted multi-gene algorithm and the BP response to pharmacotherapy. From a patient-centric outcomes perspective, this magnitude of the difference is associated with a significant reduction in heart attack and stroke risk. These findings can be applied to improving therapeutic guidance for clinicians based on known functional alterations of the protein through these genetic changes. Based on these findings, clinicians can guide therapy with knowledge specific to their patient, rather than “trial-and-error” based on population data, which will improve therapy efficacy and reduce healthcare costs, adverse events, and time to control BP. Given the retrospective nature of this trial, future randomized trials, which are controlled for the standard of care, are needed.

## Figures and Tables

**Table 1:** Functional Allele Variants Within Organ Systems, PharmGKB Clinical Annotation Level, and the Average Mean Arterial Blood Pressure Difference Between Functional and Non-Functional Allele Variants Used to Build the Mathematical Algorithm.

Target Organ	Gene	Allele	Average MAP Difference between Functional and Non-Functional	Number of Publications (PubMed)	Key References
Cardiac	Cyp2D6 *4	*rs3892097*	0 mmHg	353 *	[24,45,46,47,48,49,50,51,52,53,54,55,56,57,58,59,60]
	*ADRB1 49*	*rs1801252*	5.2 mmHg	2749 *	[48,49,50]
	*ADRB1 389*	*rs1801253*	8.7 mmHg		[49,50,51,61]
	*ADRB2 16*	*rs1042713*	3.7 mmHg	3067 *	[45,62]
	*ADRB2 27*	*rs1042714*	7 mmHg (SBP only)		[63,64]
Vascular	*ACE (I/D)*	*rs1799752*	4.7 mmHg	1377	[54,65,66]
	*AGT*	*rs699*	8.3 mmHg	2797 *	[54,67]
		*rs5051*	8 mmHg		[54,67,68,69,70]
		*rs7079*	4.3 mmHg		[70,71]
	*AGTR1*	*rs5186*	3.3 mmHg	1451	[72,73,74,75,76]
	*REN*	*rs12750834*	3.7 mmHg	1085	[30,58]
Renal	*SCNN1A*	*rs2228576*	N/A	31	[59]
	*WNK1 (a)*	*rs1159744*	3.7 mmHg	35 *	[24]
	*WNK1 (b)*	*rs2107614*	5.0 mmHg		[24]
	*WNK1 (c)*	*rs2277869*	7.0 mmHg		[24]
	*SLC12A3*	*rs1529927*	N/A	103	[39]
	*ADD1*	*rs4961*	10 mmHg	52	[40,60,77]

* Inclusive of the functional sites of that gene. MAP: mean arterial blood pressure; SBP: systolic blood pressure.

**Table 2:** Percent of Patients on the Target Drug Classes as Determined by Genotype.

Gene	Identifier	Homozygous Functional	Heterozygous	Homozygous Non-Functional
B-Blockade				
Cyp2D6 *4	*rs3892097*	32	28	31
*ADRB1*	*rs1801252*	37	19	32
	*rs1801253*	30	28	32
*ADRB2*	*rs1042713*	35	64	32
	*rs1042714*	35	45	31
ACE-Inhibitor				
*ACE (I/D)*	*rs1799752*	52	42	49
*AGT*	*rs699*	56	31	46
	*rs5051*	51	40	47
	*rs7079*	52	17	48
*AGTR1*	*rs5186*	53	15	50
*REN*	*rs12750834*	50	70	49
Angiotensin-II Receptor Blocker				
*ACE (I/D)*	*rs1799752*	24	72	22
*AGT*	*rs699*	22	60	25
	*rs5051*	21	68	24
	*rs7079*	17	44	23
*AGTR1*	*rs5186*	25	37	24
*REN*	*rs12750834*	22	89	22
Diuretic				
*SCNN1A*	*rs2228576*	44	74	36
*WNK1*	*rs1159744*	39	26	38
	*rs2107614*	42	77	39
	*rs2277869*	41	94	38
*SLC12A3*	*rs1529927*	40	30	39
*ADD1*	*rs4961*	38	12	43

Data are represented as percent (%) on target therapy as described. ACE: angiotensin-converting enzyme.

**Table 3:** Percent of Patients on the Target Drug Class as Determined by the Algorithm.

	Study Population	Match with Recommendation #1	No Match with Recommendation #1
Diuretic (%)	46.1	53.9	43.2
ACE-Inhibitors (%)	50.1	54.2	50.3
Angiotensin-II Receptor Blockers (%)	24.5	12.2	25.8
B-Blockers (%)	24.6	64.2	11.6
Other (%)	14.1	13.7	13.2

ACE: angiotensin-converting enzyme.

**Table 4:** Subject Characteristics (*N*, percent or mean ± SEM).

	Match with Recommendation #1	No Match with Recommendation #1
*N*	92	292
Age (years)	59.9 ± 1.1	61.2 ± 1.8
Height (cm)	166 ± 3	153 ± 4 *
Weight (kg)	94.6 ± 2.0	89.5 ± 1.1 *
BMI (kg/m^2^)	32.7 ± 0.8	30.8 ± 0.3 *
Past Smoker	43%	38%
Drinks (per week)	6.5 ± 0.8	5.7 ± 0.4
Exercise (min/week)	124 ± 28	193 ± 21
Self-Reported Health Status (0–4)	3.12 ± 0.06	3.16 ± 0.03

There was a statistically significant difference in height, weight, and body mass index (BMI) between patients whose therapy matched their primary genetically recommended drug class compared to patients whose therapy did not. * *p* < 0.05.

**Table 5:** Current BP Measures, Changes in BP, and BP Control Rates in Patients Who Match the Initial Recommendation vs. Those Who Do Not (percent or mean ± SEM).

	Match with Recommendation #1	No Match with Recommendation #1
Current SBP (mmHg)	128 ± 1	128 ± 1
Current DBP (mmHg)	79 ± 0	78 ± 1
Current MAP (mmHg)	95 ± 1	95 ± 1
Normotensive (JNC)	84%	85%
Normotensive (SPRINT)	27%	22%

SBP: systolic blood pressure, DBP: diastolic blood pressure, MAP: mean arterial blood pressure (calculated from SBP and DBP). JNC: The Joint National Committee BP guidelines (<140/<90). SPRINT: Systolic Blood Pressure Intervention Trial (<120/<80).

**Table 6:** Current BP Measures, Change in BP, and BP Control Rates in Patients on Monotherapy (*N*, percent or mean ± SEM).

	Match with Recommendation #1	No Match with Recommendation #1
*N*	28	129
Current SBP (mmHg)	127 ± 1	128 ± 1
Current DBP (mmHg)	79 ± 0	79 ± 1
Current MAP (mmHg)	94 ± 1	96 ± 0
ΔSBP (mmHg)	−32.8 ± 3.5	−29.6 ± 2.8 *
ΔDBP (mmHg)	−14.5 ± 3.3	−11.8 ± 2.3 *
ΔMAP (mmHg)	−21.0 ± 2.5	−16.5 ± 2.3 *
Normotensive (JNC)	92%	85%
Normotensive (SPRINT)	27%	18%

* *p* < 0.05. There was a statistically significant difference in ΔSBP, ΔDBP, and ΔMAP for patients on monotherapy whose therapy matched their primary genetically recommended drug class compared to patients whose therapy did not. SBP: systolic blood pressure, DBP: diastolic blood pressure, MAP: mean arterial blood pressure (calculated from SBP and DBP). JNC: The Joint National Committee BP guidelines (<140/<90). SPRINT: Systolic Blood Pressure Intervention Trial (<120/<80).

**Table 7:** Current BP Measures, Changes in BP, and BP Control Rates in Patients Who Match Either Recommendation #1 or Recommendation #2 (*N*, percent or mean ± SEM).

	Match with Recommendation #1 OR #2	No Match with Recommendation #1 OR #2
*N*	136	218
Current SBP (mmHg)	128 ± 1	128 ± 1
Current DBP (mmHg)	79 ± 0	79 ± 1
Current MAP (mmHg)	94 ± 1	95 ± 0
Delta SBP (mmHg)	−34.4 ± 1.7	−31.8 ± 2.3
Delta DBP (mmHg)	−15.1 ± 1.2	−13.0 ± 2.2
Delta MAP (mmHg)	−20.4 ± 1.3	−17.3 ± 2.1
Normotensive (JNC)	85%	84%
Normotensive (SPRINT)	27%	22%

SBP: systolic blood pressure, DBP: diastolic blood pressure, MAP: mean arterial blood pressure (calculated from SBP and DBP). JNC: The Joint National Committee BP guidelines (<140/<90). SPRINT: Systolic Blood Pressure Intervention Trial (<120/<80).
